# Analysis of association among clinical features and shorter leukocyte telomere length in mitochondrial diabetes with m.3243A>G mitochondrial DNA mutation

**DOI:** 10.1186/s12881-015-0238-2

**Published:** 2015-10-08

**Authors:** Mei-Cen Zhou, Rui Min, Jian-Jun Ji, Shi Zhang, An-Li Tong, Jian-ping Xu, Zeng-Yi Li, Hua-Bing Zhang, Yu-Xiu Li

**Affiliations:** Department of Endocrinology, Key Laboratory of Endocrinology, Ministry of Health, Peking Union Medical College Hospital, Beijing, 100730 China; Hongshan Traditional Chinese Medicine Hospital, Chifeng City, Inner Mongolia 024076 China; Metabolic Disease Hospital of Tianjin Medical University, Tianjin City, 300000 China; Nanyang City Center Hospital, Nanyang City, Henan 473003 China

**Keywords:** Mitochondrial diabetes, m.3243A>G mitochondrial DNA mutation, Clinical features, Leukocyte telomere length

## Abstract

**Background:**

Mitochondrial diabetes is a kind of rare diabetes caused by monogenic mutation in mitochondia. The study aimed to summarize the clinical phenotype profiles in mitochondrial diabetes withm.3243A>G mitochondrial DNA mutation and to investigate the mechanism in this kind of diabetes by analyzing the relationship among clinical phenotypes and peripheral leukocyte DNA telomere length.

**Methods:**

Fifteen patients with maternally inherited diabetes in five families were confirmed as carrying the m.3243A>G mitochondrial DNA mutation. One hundred patients with type 2 diabetes and one hundred healthy control subjects were recruited to participate in the study. Sanger sequencing was used to detect the m.3243A>G mitochondrial DNA mutation. The peak height G/A ratio in the sequence diagram was calculated. Real-time polymerase chain reaction (PCR) was used to measure telomere length.

**Results:**

The patients with mitochondrial diabetes all had definite maternally inherited history, normal BMI (19.5 ± 2.36 kg/m^2^), early onset of diabetes (35.0 ± 14.6 years) and deafness. The peak height G/A ratio correlated significantly and negatively with the age at onset of diabetes (≦25 years, 61.6 ± 20.17 %; 25–45 years, 16.59 ± 8.64 %; >45 years, 6.37 ± 0.59 %; *p* = 0.000). Telomere length was significantly shorter among patients with mitochondrial diabetes and type 2 diabetes than in the control group (1.28 ± 0.54 vs. 1.14 ± 0.43 vs. 1.63 ± 0.61; *p* = 0.000). However, there was no significant difference between patients with mitochondrial diabetes and those with type 2 diabetes. There was no correlation between telomere length and the peak height G/A ratio.

**Conclusion:**

Deafness with definite maternal inheritance and normal BMI, associated with elevated blood lactic acid and encephalomyopathy, for the most part, suggest the diagnosis of mitochondrial diabetes . The peak height G/A ratio could reflect the spectrum of age at onset of the disease. Telomere length was shorter in patients with mitochondrial diabetes and those with type 2 diabetes, which suggests that the shorter telomere length is likely involved in the pathogenesis of diabetes but is not specific for this kind of diabetes.

## Background

Mitochondrial diabetes is a kind of rare diabetes caused by monogenic mutation in mitochondria, which was first described in 1992, results from an A to G substitution at nucleotide position 3243 (m.3243A>G mitochondrial DNA mutation) of the mitochondrial gene encoding tRNA^Leu^ [1–4]. Eighty-five percent of mitochondrial diabetes cases are associated with m.3243A>G mitochondrial DNA mutation [5], and although other mitochondrial DNA point mutations have been associated with mitochondrial diabetes, these occurrences are extremely rare [6]. Because mitochondria are distinct organelles in the cytoplasm, its inheritance is only maternal, and mitochondrial DNA mutations have different heteroplasmy levels that vary among different tissues in a single individual; hence, there is a wide range of clinical phenotypes and mitochondrial diabetes is frequently misdiagnosed as either type 1 or type 2 diabetes, depending on the clinical presentation [5].

DNA telomeres, which are specific DNA-protein structures in the terminal regions of chromosomes, are a biomarker of biological aging and can reflect the lifespan of cells [7]. Telomere shortening has been observed in subjects with type 2 diabetes or impaired glucose tolerance, for example, in Chinese [8], Afro-Caribbean [9], Arabic and Caucasian [10] populations. Many factors are involved in telomere shortening; for instance, reactive oxidative species (ROS) as by-products of mitochondrial oxidative phosphorylation (OXPHOS) play an important role in telomere shortening [11–13]. Mutations in mitochondrial DNA have an impact on the mitochondrial function of the pancreas among patients with mitochondrial diabetes. However, whether the change in the mitochondrial function in patients with mitochondrial diabetes can affect DNA telomere length is still unknown. In this study, we hypothesize that telomere length shortening exists in the beta cells of mitochondrial diabetes, which can shorten the lifespan of beta cells. Exploring the pathogenesis by investigating the relationship between changes in mitochondrial function and telomere length can provide new insight.

The present study aimed to summarize the profiles of clinical phenotypes in fifteen patients with mitochondrial diabetes from five different families and to investigate the mechanism of mitochondrial diabetes by analyzing the relationship among clinical phenotypes and peripheral leukocyte DNA telomere length in blood.

## Methods

### Study subjects

According to the inclusion criteria for this study, forty subjects with diabetes in Peking Union Medical College were included between 2007 and 2014. The inclusion criteria were as follows: ① age at the diagnosis of diabetes ≤ 40 years old; ② BMI ≤ 24 kg/m^2^; ③ negative autoimmune diabetes antibodies: insulin autoantibody IAA (−), islet cell antibody ICA (−), glutamate decarboxylase antibody GAD (−), and protein-tyrosine-phosphatase antibody IA2 (−); ④ maternal family history of diabetes; ⑤ deafness; ⑥ elevated serum lactic acid level; and ⑦ encephalomyopathy. Items ①,②,③,④are necessary items, with or without items ⑤,⑥,⑦. Five patients were confirmed as carrying the m.3243A>G mitochondrial DNA mutation, these patients were defined as mitochondrial diabetes. By further screening the family members of the five probands, a total of fifteen patients with mitochondrial diabetes (age range: 17 ~ 68 years-old) were confirmed. One hundred patients with type 2 diabetes (age range: 22 ~ 67 years-old) and one hundred healthy individuals without diabetes (age range: 19 ~ 65 years-old) were recruited to participate in the study and were matched according to age and gender. The study protocol was approved by the Ethics Committee of Peking Union Medical College Hospital. The subjects signed an informed consent form and all the subjects provided written informed consent for the publication of their clinical details. We also obtained the written informed consent from the guardians of the only one subjects who was 17 years-old. The study protocol was approved by the Ethics Committee of Peking Union Medical College Hospital.

### M.3243A>G mitochondrial DNA mutation screening method

Genomic DNA from peripheral lymphocytes was isolated using the QIAamp DNA Mini Kit (QIAGEN, Germany). The PCR assay was designed to detect the mt 3243A to G mutation using a direct PCR. The following two primers were used in one reaction tube:

- Forward: 5′-AGCGCCTTCCCCCGTAAATGA-3′,
- Reverse: 5′-AGAATGATGGCTAGGGTGACTTC-3′.

The primers were designed using Oligo 6.0. Nucleotides 3160–3620 of mt DNA were amplified. Each PCR product was segregated using agarose gel electrophoresis and purified using the gel extraction method before being sequenced in an ABI3730xl sequencer using the forward PCR primer. The sequencing data were aligned using BLAST Search Genome (http://genome.ucsc.edu). Each sample was sequenced twice.

### The peak height G/A ratio in the sequence diagram

**Fig. 1:**
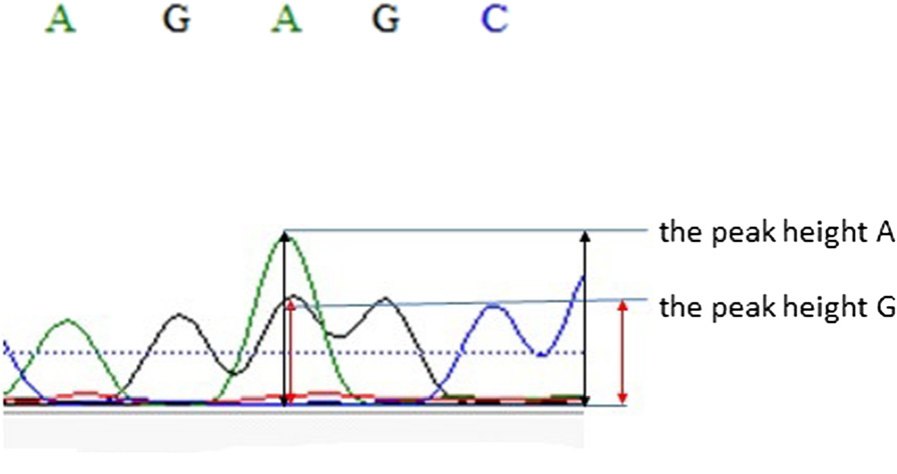
The peak height G/A ratio was defined as the peak height A was devided by the peak height G

The peak height G/A ratio in the sequence diagram was calculated according to the mean value of the sequences (Each sample was sequenced twice). The peak height G/A ratio was defined as the peak height A was divided by the peak height G as show in Fig. 1.

### Leukocyte DNA telomere length measurement

Telomere length was determined as the relative ratio of telomere repeat copy number to the single copy number (T/S) using the novel monochrome multiplex quantitative PCR protocol described by Cawthon [14]. Telomere primer sequences were as follows:

- telg : 5′-ACACTAAGGTTTGGGTTTGGGTTTGGGTTTGGGTTAGTGT-3′,
- telc : 5′-TGTTAGGTATCCCTATCCCTATCCCTATCCCTATCCCTAACA-3′,

and albumin was employed as the single copy gene reference using primers modified with the addition of 5′-GC clamp to shift their melting temperature:

- albu : 5′-CGGCGGCGGGCGGCGCGGGCTGGGCGGAAATGCTGCACAGAATCCTTG-3′;
- albd : 5′-GCCCGGCCCGCCGCGCCCGTCCCGCCGGAAAAGCATGGTCGCCTGTT-3′.

The reagent components and final concentrations were 900 nM each primer (IDT), 1 × AmpliTaq Buffer II, 3 mM MgCl_2_, 0.2 mM per dNTP, 1 mM DTT, 1 M betaine, 0.75 × SYBR Green I and 0.625U AmpliTaq Gold DNA polymerase. Human genomic DNA samples, 5 ng to 70 ng, were used to generate two standard curves for each PCR plate (five concentrations with a high level of 150 ng and a low level of 1.85 ng per reaction). Thermal cycling: 1 cycle of 15 min at 95 °C; 2 cycles of 15 s at 94 °C, 15 s at 49 °C; and 32 cycles of 15 s at 94 °C, 10 s at 62 °C, 15 s at 74 °C with signal acquisition, 10 s at 84 °C, and 15 s at 88 °C with signal acquisition. Bio-Rad CFX Manger software automatically estimated the value for each sample T (telomere) and S(single copy gene) using standard curve. Standard curve efficiencies for both primer sets were above 90 %, and regression coefficients were at least 0.99 in all PCR runs. The within-plate and between-plate % coefficient of variation(% CV), which is based on the ratio of the standard deviation across replicates to the mean, were 18 % and 7 %, respectively. For study samples, the with-plate % CV ranged from 8.2–14.3 %.

### Statistical analyses

The statistical analyses were performed using SPSS 17.0. Parameters not normally distributed were transformed. The variables were expressed as the mean ± SD. Statistical analyses were performed with ANOVA followed by Bonferroni’s post hoc pairwise comparisons. The multivariable linear regression analysis was used to test the relationship between variables and the risk factors.

## Results

**Fig. 2:**
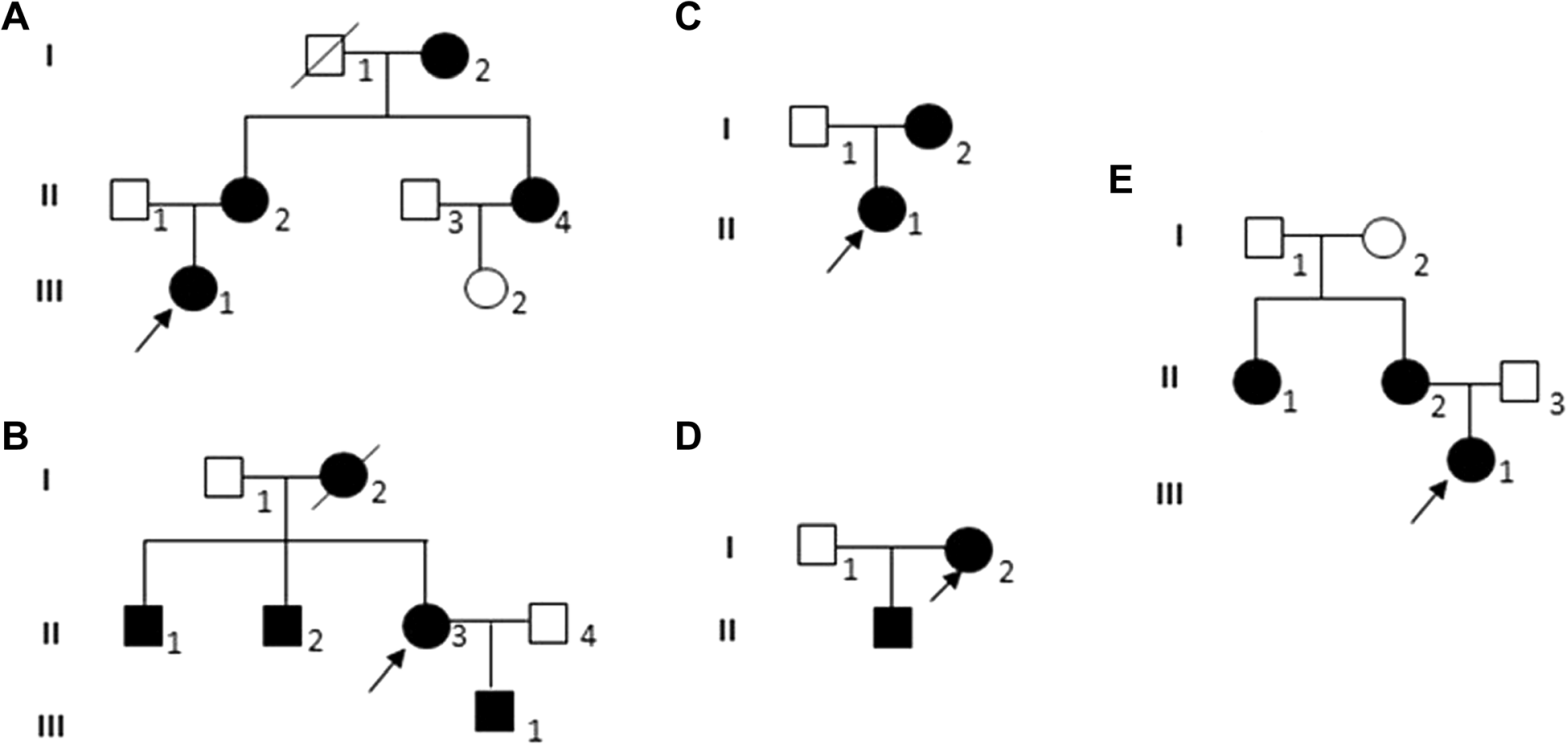
**a** Pedigree of family I. **b** Pedigree of family II. **c** Pedigree of family III. **d** Pedigree of family IV. **e** Pedigree of family V.   Show the patient with maternally inherited diabetes and deafness,   Show family member without the disease.  Shows the proband in the family. II1 in family II and II1 in family IV have MELAS and mitochondrial diabetes. III1 in family II has mt DNA 3243 mutation, although he did not have symptoms of mitochondrial diabetes

1. **Clinical features in mitochondrial diabetes**
  Five patients with mitochondrial diabetes from five different families were confirmed by m.3243A>G mitochondrial DNA mutation screening from forty suspected patients with mitochondrial diabetes; screening rate was 12.5 %. In the other maternal members of the five patients from the five families, there were eleven subjects with m.3243A>G mitochondrial DNA mutation among the eleven subjects, ten subjects suffered from mitochondrial diabetes, only one subject had mutation without any clinical presentation. Therefore, in total, there were fifteen patients with mitochondrial diabetes in the five families (Fig. 2). The mean age at the diagnosis of diabetes the five probands was 23.1 ± 8.2 years, and the onset of impaired hearing loss was before the diagnosis of diabetes. The five probands were all female; two of them had oligomenorrhea, their follicle stimulating hormone(FSH) and luteinizing hormone(LH)level was below the normal range (Table 1). At the onset of mitochondrial diabetes, the mean age of the fifteen patients was 35.0 ± 14.6 years, their BMI was 19.5 ± 2.36(kg/m^2^), and one patient presented acutely with ketoacidosis-related type 1 diabetes, while the others presented insidiously with symptoms similar to type 2 diabetes. The general and specific clinical features of the 15 patients with mitochondrial diabetes are shown in Tables 2 and 3, respectively.

**Table 1:** Blood gonadal hormone levels in 2 patients with mitochondrial diabetes combined with oligomenorrhea

	Age (year)	BMI (kg/m^2^)	FSH (mIU/L)	LH (mIU/L)	T (nmol/L)	E2 (pmol/L)
III1 (P:I)	18	17.4	5.5	3.9	1.7	355.25
II1 (P:III)	26	21.7	1.2	0.21	3	28.7

*P* I-V represent family I-V, *Age* age at onset of oligomenorrhea, *FSH* follicle stimulating hormone, *LH* luteinizing hormone, *T* testosterone, *E2* estradiol

**Table 2:** General clinical features of mitochondrial diabetes

	Mitochondrial diabetes (*n* = 15)
Age (year)	35.0 ± 14.6
Gender (F/M)	12:3
Disease Duration (year)	4.00 ± 2.54
BMI (kg/m^2^)	19.5 ± 2.36
HbA1c (%)	6.42 ± 1.01
Fasting blood glucose (mmol/L)	7.75 ± 2.03
Fasting insulin (μIU/ml)	5.23 ± 3.26
Fasting C-peptide (ng/ml)	0.53 ± 0.37
HOMA-IR	1.63 ± 0.81
HOMA-IS	0.03 ± 0.02
Diabetes treatment	7:8
(Insulin: oral hypoglycemic medication)	(5 probands all received insulin therapy)

*BMI* body mass index, *HOMA-IR* fasting plasma glucose (mmol/L) *fasting insulin (μIU/ml)/22.5, *HOMA-IS* 1/fasting plasma glucose (mmol/L) *fasting insulin (μIU/ml)

**Table 3:** Specific clinical features in mitochondrial diabetes (*n* = 15)

	Family member	Age at diagnosis (year)	BMI (kg/m2)	Autoimmune diabetes antibodies	Deafness	Fasting/postprandial elevated blood lactate	Encephalomyopathy	The peak height G/A ratio%
1	II1 (P:I)	68	18.4	ND	+	ND	-	5.9
2	II2	49	23.4	IAA (−)、ICA (−)、GAD (−)	+	ND	-	14
3	II4	48	16.8	IAA (−)、ICA (−)、GAD (−)	+	ND	-	14.9
4	III1	18	18.7	IAA (−)、ICA (−)、GAD (−)	+	-	-	83.3
5	I2 (P:II)	25	17.4	ND	+	ND	-	31.6
6	II1	31	21.2	ND	+	+	+	29.1
7	II2	43	20.1	ND	+	ND	-	8.2
8	II3	38	19	IAA (−)、ICA (−)、GAD (−)	+	ND	-	27.6
9	I2 (P:III)	43	18.4	ND	+	ND	-	8
10	II1	17	18.3	IAA (−)、ICA (−)、GAD (−)	+	ND	-	65
11	I2 (P:IV)	33	16.7	IAA (−)、ICA (−)、GAD (−)	+	-	-	29
12	II1	24	21.7	ND	+	+	+	27.1
13	II1 (P:V)	26	24.9	ND	+	ND	-	34.5
14	II2	46	19.1	ND	+	ND	-	15.2
15	III1	17	18.4	IAA (−)、ICA (−)、GAD (−)	+	ND	-	63.6

For the structure of the five- mitochondrial diabetes pedigree, see Fig. 1. P, I-V represent family I-V.;ND represents undetected;-, negative; +, positive. The peak height G/A ratio, m.3243A>G mitochondrial DNA mutation peak height G to A ratio
2. **Comparison of telomere length among patients with mitochondrial diabetes and type 2 diabetes and healthy controls**
  The ages were matched among the three groups, and the duration of diabetes was matched between those with mitochondrial diabetes and those with type 2 diabetes. There was a significant difference in BMI among the three groups. Peripheral blood leukocyte DNA telomere length was significantly shorter in patients with mitochondrial diabetes and type 2 diabetes than in healthy controls; however, there was no significant difference between those with mitochondrial diabetes and those with type 2 diabetes (Table 4).

**Table 4:** Comparison of age, duration of diabetes, BMI, and telomere length

	Mitochondrial diabetes (*n* = 15)	T2DM (*n* = 100)	Healthy controls (*n* = 100)	*P*
Age (year)	35.2 ± 19.1	42.2 ± 14.3	38.3 ± 10.3	0.070
Duration of diabetes (year)	4.0 ± 2.7	4.8 ± 3.5	——	0.440
BMI (kg/m^2^)	19.5 ± 2.36	24.3 ± 2.4	21.2 ± 1.4	0.000***
Telomere length	1.28 ± 0.54	1.14 ± 0.43	1.63 ± 0.61	0.000***

***p < 0.001. There was a significant difference in BMI and telomere length among the three groups
3. **Correlation analysis between m.3243A>G mitochondrial DNA mutation (the peak height G/A ratio) and onset age of mitochondrial diabetes**
  The peak height G/A ratio in the sequence diagram was calculated. The peak height G/A ratio was significantly different according to the age at the diagnosis of diabetes (≤25 years, 25–45 years, and >45 years) (Table 5) and was negatively associated with the age at the diagnosis of diabetes after adjusting the age, BMI (*r* = −0.891; *p* < 0.001).

**Table 5:** m.3243A>G mitochondrial DNA mutation the peak height G to A ratio among different ages at onset of mitochondrial diabetes

Age of onset (year) (*n* = 15)	≤25 (*n* = 5)	25–45 (*n* = 6)	>45 (*n* = 4)
The peak height G to A ratio	61.60 ± 20.17**	16.59 ± 8.64**	6.37 ± 0.59**

**p < 0.01. There was a significant difference among different ages at onset of mitochondrial diabetes
4. **Correlation between telomere length and m.3243A>G mitochondrial DNA mutation (the peak height G/A ratio) and diabetes-related parameters**
  There was no significant relationship between peripheral blood leukocyte DNA telomere length and the peak height G/A ratio after adjusting age fasting glucose, insulin, C-peptide, HbA1c, HOMA-IR, and HOMA-IS. Fasting glucose, insulin, C-peptide, HbA1c, HOMA-IR, and HOMA-IS were not significantly related to DNA telomere length (Table 6).

**Table 6:** Correlation analysis between leukocyte telomere length and FBG, HbA1c, fasting C-peptide, fasting insulin, HOMA-IR index and HOMA-IS index, and the peak height G to A ratio

	Correlation coefficient r	P
Fasting blood glucose	0.282	0.374
HbA1c	0.014	0.965
Fasting C-peptide	0.332	0.292
Fasting insulin	0.243	0.463
HOMA-IR	−0.127	0.694
HOMA-IS	0.253	0.427
the peak height G to A ratio	−0.156	0.646

All data were analyzed after correction for age and duration of disease. All data were analyzed using Pearson correlation analysis

## Discussion

Patients with mitochondrial diabetes generally exhibit heteroplasmy levels of between 1 % and 40 % with regard to m.3243A>G mitochondrial DNA mutation in the blood [6]; however, heteroplasmy levels may vary in different tissues in a single individual. Although blood generally contains the lowest heteroplasmy levels [15, 16], detecting m.3243A>G mitochondrial DNA mutation in blood is used as a simple and widely available screening method. The present study screened m.3243A>G mitochondrial DNA mutation in forty patients with suspected mitochondrial diabetes, five patients were confirmed to have the disease; the screening rate was 12.5 %, which was considerably higher than 1.69 % (the screening rate in patients diagnosed with type 2 diabetes, as reported by Xiang et al. [17]). This result suggested that targeted screening in patients with suspected mitochondrial diabetes (combined with early onset of diabetes and normal BMI) could improve the diagnosis of mitochondrial diabetes.

Mitochondrial diabetes is frequently misdiagnosed as either type 1 or type 2 diabetes depending on the age of the patients and the mode of presentation. In the present study, patients with mitochondrial diabetes had a definite maternal history of diabetes; four out of five probands represented the second or third generation with diabetes in the family, which suggests that maternal history of diabetes plays an important role in the diagnosis. The mean age at the onset of diabetes was 35 years-old, ranging from 17 years to 68 years; however, the age at onset for the proband in each family was relatively young, with normal BMI, accompanied by different degrees of deafness in the high-frequency domain. In the study, all the patients with mitochondrial diabetes carrying m.3243A>G mitochondrial DNA mutation had deafness, suggesting the strong association between mitochondrial diabetes and hearing loss, which was in accordance with Mancuso M, et al. [18]. Several female patients had oligomenorrhea caused by idiopathic hypogonadotropic hypogonadism, which suggests that mitochondria dysfunction could be involved in pituitary function, leading to idiopathic hypogonadotropic hypogonadism. In this study, even in the same family, the clinical presentations were different; the progress of diabetes was not consistent; several patients presented acutely with ketoacidosis-related type 1 diabetes; and other patients presented insidiously with symptoms similar to type 2 diabetes. Although siblings in the same families had the same m.3243A>G mitochondrial DNA mutation, the clinical manifestations were different. In family II, the sister presented with diabetes and deafness, whereas the brother had diabetes and deafness and also presented with MELAS (mitochondrial encephalomyopathy with lactic acidosis and stroke-related episodes). Therefore, to properly diagnose mitochondrial diabetes, understanding the profiles of the clinical presentations of the disease is very important.

Heteroplasmy levels lead to varying clinical phenotypes in different tissues [19, 20]. In patients with mitochondrial diabetes, mitochondrial dysfunction in beta cells causes insulin secretion disorder [21, 22], imbalance of ion concentrations, and cell death within the vascular stria that leads to reduction in sound transduction [23]. Direct PCR and sequencing analysis (Sanger sequencing) are widely available and simple methods for detecting m.3243A>G mitochondrial DNA mutation. Although the peak height G to A ratio does not represent the real value of heteroplasmy levels, it is widely used for detecting point mutation in disease diagnosis. This study found that the peak height G to A ratio was negatively associated with age at the onset of mitochondrial diabetes, that the ratio was higher, and that age at the onset of the disease was younger. The peak height G to A ratio depended on m.3243A>G mitochondrial DNA mutation heteroplasmy levels, according to the research conducted by Laloi-Michelin et al. [23]. The results suggested that although Sanger sequencing is not a quantitative method compared with site-specific quantitative PCR, it could be a simple method for indicating, to some extent, the degree of severity of mitochondrial diabetes in clinical work.

Different m.3243A>G mitochondrial DNA mutation heteroplasmy levels result in varying energy metabolism disorders of mitochondria [24]. The mechanism of diabetes is complicated. Mitochondrial diabetes caused by monogenic mutation is an ideal model for investigating the pathogenesis of diabetes. Mitochondria, which are an essential component of energy production through oxidative phosphorylation (OXPHOS), have a close relationship with cellular senescence. Mitochondria generate a large amount of reactive oxidative species (ROS) as a toxic by-product of OXPHOS, which plays an important role in telomere shortening [25]. Mitochondrial dysfunction caused by DNA mutations is observed in mitochondrial dysfunction in the pancreas has an impact on beta cell secretion function in patients with mitochondrial diabetes [21, 22]. Telomere length can most likely be associated with the lifespan of beta cells, which is related to the secretion function of beta cells. To the best of our knowledge, this study is the first to investigate telomere length in patients with mitochondrial diabetes. This study found that compared with healthy controls, telomere length was shorter in patients with mitochondrial diabetes, which indicated that telomere length shortening likely participates in the pathogenesis of mitochondrial diabetes; however, when compared with type 2 diabetes, there was no significant difference; thus, telomere length is not specific to mitochondrial diabetes. This study investigated the correlation between telomere length, peak height G to A ratio, and blood glucose condition and noted that there was no significant difference between these variables. These observations suggest that telomere length is affected by many factors (higher BMI [26]) other than ROS; however, we cannot rule out the impact of the small sample size in the present study.

In this study, the number of patients with mitochondrial diabetes using insulin and oral hypoglycemic medications is equal, but the five probands all received insulin treatment at the very beginning of diabetes, which indicated worse beta cell function. An energy metabolism disorder exists in mitochondrial diabetes and is usually accompanied by elevated blood lactate levels; thus, to avoid lactic acidosis, oral hypoglycemic medications, such as metformin, should not be used [26]. In view of this risk, it is important to differentiate mitochondrial diabetes from type 1 or type 2 diabetes.

## Conclusion

Deafness with definite maternal inheritance, normal BMI, and elevated blood lactic acid and encephalomyopathy, for the most part, suggests the diagnosis of mitochondrial diabetes. According to Sanger sequencing, the peak height G/A ratio could reflect the spectrum of age at the onset of the disease and might therefore provide a prediction regarding the age of onset in particular cases. Telomere length was shorter in mitochondrial diabetes and type 2 diabetes, which suggests that telomere length shortening is likely involved in the pathogenesis of diabetes; however, it was not specific for mitochondrial diabetes. Our study also had some limitations, the study included very limited patient population and the heteroplasmy levels were detected in blood instead of urine. Therefore, in future, further, larger and better designed studies focused on the topic are still needed to draw more definitive conclusions.
